# Concomitant Obstructive Sleep Apnoea in Patients with Meniere's Disease: A Case Report and Literature Review

**DOI:** 10.1155/2021/5592611

**Published:** 2021-03-23

**Authors:** Wong Kein Low, Esther Jiayi Lim

**Affiliations:** ^1^Novena Ent-Head & Neck Surgery Specialist Centre, 04-21/22/34, Mount Elizabeth Novena Medical Centre, 38 Irrawaddy Road, Singapore; ^2^Duke-NUS Graduate Medical School, 8 College Road, Singapore

## Abstract

Meniere's disease (MD) is a condition characterised by fluctuating and progressive hearing loss, aural fullness, tinnitus, and intermittent attacks of vertigo. The disabling vertigo symptoms can be controlled in most patients by lifestyle changes and medications such as diuretics. Should standard medical therapy fail, the patient may require surgery in order to control the disease, but such surgical procedures can be functionally destructive. Obstructive sleep apnoea syndrome (OSAS) is common, especially in people who are grossly overweight. Up to 15% of patients with MD may have concomitant OSA. Unless the OSA is well controlled, such patients may continue to experience MD symptoms despite receiving adequate standard medical therapy for MD. Moreover, MD patients may experience insomnia as a result of vertigo and/or tinnitus where sedatives are indicated. The use of sedatives with muscle relaxant properties may inadvertently further aggravate OSA resulting in a vicious cycle of symptoms. Symptoms suggestive of concomitant OSA must be proactively sought as these patients do not necessarily exhibit the obvious phenotypic features of OSA. This is especially so in Asians where OSAS is commonly observed in people who are not overly obese. We report a case of a female patient who presented with recalcitrant MD disease and was later found to have concomitant OSA. The relevant literature will be reviewed, and learning points will be discussed from the perspective of the otologist/neurotologist. The clinician must always be mindful of the existence of concomitant “silent” OSAS as this impacts the management of patients with MD.

## 1. Introduction

Meniere's disease (MD) is a condition characterised by fluctuating and progressive hearing loss, aural fullness, tinnitus, and intermittent attacks of vertigo. Although the aetiology of MD is largely unknown, the pathology is the result of hydropic distension of the endolymphatic system [1]. Various factors such as stress are known to trigger or contribute to MD [2, 3].

Obstructive sleep apnoea syndrome (OSAS) is a sleep-related breathing disorder where episodes of apnoea/hypopnea occur during sleep due to a collapse of the upper airway. The direct consequences of the collapse are intermittent hypoxia and hypercapnia, recurrent arousals, and increase in respiratory efforts, leading to secondary sympathetic activation, oxidative stress, and systemic inflammation [4]. The cardiovascular complications of OSA are well documented [5], but the neurotological consequences have only been studied recently [6]. A mainstay of treatment for OSA is continuous positive airway pressure (CPAP), which corrects the hypoxia and associated body stresses.

In recent years, there have been some reports linking MD and OSA [6–10]. Up to 15% of patients with MD have been found to have concomitant OSAS [11]. It is important to identify this subset of patients because unless the OSA is well controlled, such patients may continue to experience MD symptoms despite receiving adequate standard medical treatment for MD. Moreover, MD patients may experience insomnia from the vertigo and/or tinnitus where sedatives are indicated. The use of sedatives with muscle relaxant properties may inadvertently further aggravate OSAS resulting in a vicious cycle of symptoms. It is noteworthy that identification of OSAS in patients with MD may not be forthcoming as these patients do not necessarily exhibit obvious phenotypic features such as gross obesity that characterise OSAS. This is, especially so in Asians [6].

We report a case to illustrate the relationship between MD and OSA and highlight some of the challenges encountered. The relevant literature will be reviewed and discussed from the perspective of the otologist/neurotologist.

## 2. Case Report

A 53-year-old Chinese female was first seen by us in September 2016 with an 18-month history of recurrent episodes of vertigo associated with nausea and vomiting. Each of these vertiginous episodes typically lasted for 7-8 hours and appeared to be aggravated by stress. She also experienced fluctuating tinnitus, hearing loss, and aural fullness in the right ear. She had a history of rhinitis.

She also complained of headaches which were usually located at the back or the top of the head. Often occurring after sleep, they were described as dull in nature and not pulsatile. The headaches were mild to moderate in severity which did not interfere nor prohibit daily activities. They might be aggravated by stress but not by physical activities. She did not have photophobia, phonophobia, nor visual auras. The fluctuating aural symptoms (but not the headaches) occurred around the time of the vertiginous episodes.

The patient was anxious about her symptoms which were affecting her quality of life and work. She had difficulty sleeping, which she attributed to her MD symptoms. It was noteworthy that she did not offer at presentation a history of snoring, apneic episodes during sleep, or any history suggestive of OSAS.

**Figure 1:**
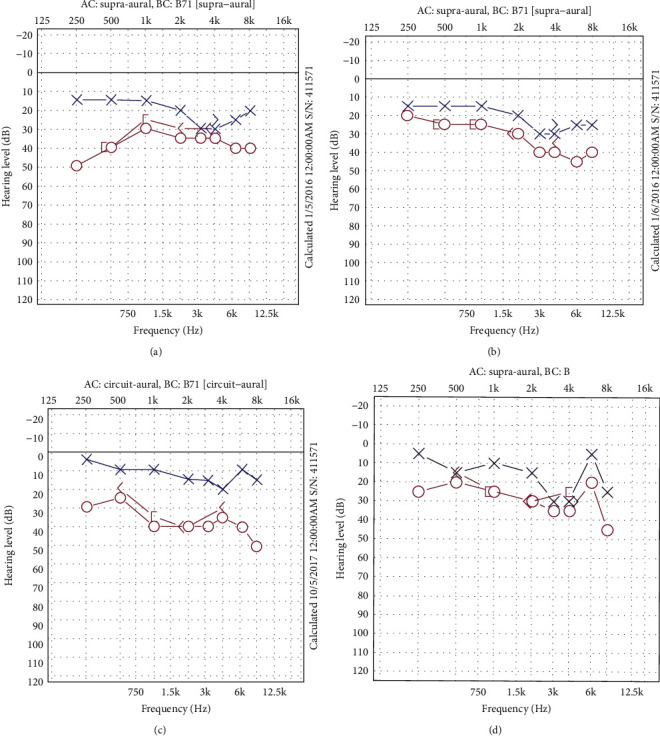
Fluctuating hearing levels affecting mainly the low- to midfrequencies in the right ear which corresponded to the fluctuating aural symptoms in the same ear. Audiogram done during the initial episode (a), after treatment with medications (b), during a subsequent episode (c), and after treatment with CPAP (d).

With a body weight of 57 kg and a height of 157 cm, her body mass index (BMI) was 23.1 kg/m^2^. She did not have any craniofacial features to suggest OSAS. Neurootological assessment was unremarkable. Audiogram showed sensorineural hearing loss in the right ear, especially in the lower frequencies (Figure 1). MRI scan of the brain was normal.

Based on the amended diagnostic criteria (2015) for MD by the American Academy of Otolaryngology-Head and Neck Surgery (AAO-HNS), a clinical diagnosis of MD was made [12]. This was further substantiated by audiometric documentation of fluctuating low-to-midfrequency hearing levels at different time points (Figure 1).

Lifestyle changes such as a low-sodium and low-caffeine diet were advised. A course of Apotriazide was prescribed for each episode of attacks with betahistine being used as maintenance. However, she continued to have recurrent vertiginous attacks and fluctuating right aural symptoms. She still experienced difficulty sleeping and was referred to a psychiatrist who prescribed clonazepam and Valdoxan. The family sought a second opinion from an ear specialist in the USA who concurred with the diagnosis of MD.

After almost 1 year of standard medical therapy, her MD was still not adequately controlled with recurring bouts of vertigo. The patient had a supportive spouse who was keen to exclude any comorbidity that could have contributed to the disease. Having observed her sleeping patterns, he suspected that she might have OSAS. A sleep study was done in September 2017 which confirmed severe sleep apnoea with an apnoea-hypopnea index (AHI) of 35.6 events per hour. The results of the study suggested that the sleep apnoea was obstructive and not central in origin. Upon direct questioning, she admitted to having regular snoring with apneic episodes during sleep. Nasopharyngoscopy showed narrowing of the hypopharyngeal space. Hypertrophic inferior turbinates and deviated nasal septum were also observed. The tonsils were, however, not enlarged.

Upon diagnosis of OSA, she was started on continuous positive airway pressure (CPAP). Since then, she had complete resolution of vertigo as well as headaches over a follow-up period of 28 months. She had continued with betahistine (but not Apotriazide) as maintenance for about 15 months after starting CPAP and used only CPAP thereafter.

## 3. Discussion

The diagnosis of MD is a clinical one. The AAO-HNS issued a set of amended diagnostic criteria for MD in 2015 (Table 1), which were formulated together with the Classification Committee of the Barany Society, the Japan Society for Equilibrium Research, the European Academy of Otology and Neurotology, and the Korean Balance Society [12]. As shown in Table 1, our patient possessed the clinical features that defined definite MD. Our patient also had audiometric documentation of fluctuating hearing levels involving mainly the low- to midfrequencies which further supported the diagnosis of MD (Figure 1).

Episodes of vertigo developing in a patient with headaches raise the possibility of vestibular migraine as the cause.  Table 2 outlines the diagnostic criteria of vestibular migraine issued by the Barany Society and the International Headache Society [13]. Based on this set of criteria, our patient clearly did not have vestibular migraine (Table 2). Instead, the headaches experienced by our patient were more likely a result of her OSA [14]. This was substantiated by the fact that her headaches completely resolved after CPAP treatment.

The prevalence of MD has been reported to be between 12 and 46 per 100,000, with a geographical variation [7]. The otologist/neurotologist routinely manages MD patients, and standard medical therapy includes lifestyle changes such as dietary salt restriction and medications such as diuretics, migraine prophylaxis, and steroid therapy [15]. Should adequate standard medical therapy fail to control frequent disabling vertigo, more effective but potentially functionally destructive treatment modalities such as gentamicin ablation, vestibular neurectomy, and labyrinthectomy may be indicated [1]. Such procedures will result in total loss of vestibular function in the operated ear, and if the opposite ear is subsequently affected by MD, the patient may end up with significant loss of vestibular function in both ears which can be functionally disabling. This is because the vestibular function in an ear affected by MD can possibly be lost as a result of the disease itself. In this respect, it is important to note that MD not uncommonly affects both ears over time. In one study, 14% of patients with unilateral MD became bilateral over an average follow-up period of 7.6 years (SD = 7.0 years) [16]. Hence, any comorbidity, which if treated can lead to better outcomes with conservative treatment, should be identified and managed. As illustrated by the present case report, concomitant OSA is potentially such a comorbidity that has often been underappreciated.

The prevalence of clinically significant OSAS in the general adult population has been reported to be at least 8.5% [7]. In a longitudinal study, the association between MD and OSA patients was studied based on a nationwide 9-year longitudinal cohort database of 1,025,340 South Korean patients [9]. In this study, no overall association between OSA and MD was observed. However, in a subgroup analysis, female and middle-aged (45–64 years) patients with OSAS were independently associated with a two-fold higher incidence of subsequent MD compared to those without OSA. These findings are consistent with the profile of our patient in the present case report. Among patients with MD, the prevalence of concomitant OSA has been reported to be up to 15% [11].

The patient in this case report, who is Chinese with a BMI of 23.1 kg/sq m, highlighted an important learning point. Concomitant OSAS in MD patients is not necessarily obvious to the clinician and can be easily missed. Nakayama and Kabaya also reported 2 patients who did not present with symptoms suggestive of sleep disturbances until when asked [6]. The 2 patients had moderate OSA but were not excessively overweight. Racial differences in craniofacial structures exist, and Asians may suffer from significant OSAS with lower weight gains as compared to Western patients [17–19]. In a larger series of 20 Asian patients with MD and OSAS, the body mass index (BMI) was found to range from 20.7 to 28.5 with an average of only 23.5 kg/sq m [7]. In a case report, Pisani et al. reiterated that the clinician must be mindful of the coexistence of “silent” OSAS among MD patients, especially those who were not responsive to standard medical treatment [8].

The importance of identifying OSAS in MD patients is twofold. Firstly, as highlighted by Nakayama and Kabaya, failure to identify OSA can result in the use of medications than can adversely affect the outcomes [6]. MD patients are often prescribed sedatives such as benzodiazepines to relieve anxiety and insomnia. The use of such sedatives which have muscle relaxant properties may inadvertently further aggravate OSA resulting in a vicious cycle of symptoms. This could have been the case for our patient in this case report.

Secondly, it may not be possible to medically control the symptoms of MD unless the comorbidity of OSA is addressed. OSA is a source of body stress and can be a potent aggravating factor of the symptoms of MD. As illustrated by our patient, the symptoms of MD were eventually controlled by effective treatment of OSA using CPAP [6, 7, 10].

The pathophysiology linking OSA to MD remains controversial. Several reports have suggested that vascular occlusion played an important role in MD. In a transcranial Doppler sonographic study, compromise of the vertebrobasilar vascular circulation was found in 28% of MD patients [20]. Histologic studies of patients with MD revealed total or partial obstruction of small vessels around the endolymphatic sac [21]. This was substantiated by the observation that cochlear blood flow was impaired in experimentally induced endolymphatic hydrops in guinea pigs [22]. In fact, the cornerstone of the treatment protocol for MD patients at the University of Colorado was identification and control of disorders that impair cerebral and inner ear vascular perfusion [15].

Vascular occlusion is also closely associated with OSA. Night-time hypoxaemia from sleep apnoea can result in sympathetic overactivity during waking hours, endothelial damage, platelet aggregation, and chronic systemic and pulmonary hypertension [5]. Migraine, perhaps through the mechanism of ischaemia from vasospasm, frequently occurred in OSA particularly in the younger patients [15]. As hypertension and migraine are themselves risk factors for cerebrovascular and cardiovascular diseases, sleep apnoea is both a primary vascular risk factor and a secondary risk factor through these allied conditions [10].

Intermittent hypoxia itself could also contribute to the cardiometabolic consequences of OSA. The molecular pathways and cellular interactions involved were recently studied by Arnaud et al. [23]. The authors found that intermittent hypoxia resulted in sympathetic activation, low-grade inflammation, oxidative stress, and endoplasmic reticulum stress with the hypoxia-inducible factor-1 transcription factor and mitochondrial functional changes playing a role.

Oxidative stress could result in a breakdown of the blood-labyrinthine barrier, and the blood-labyrinthine barrier is critical in the maintenance of inner ear homeostasis [24]. Kim et al. believed that vascular occlusion and intermittent hypoxic events in OSA led to enhanced oxidative stress in the inner ear which could result in MD [9]. The symptoms of MD in such patients could then be possibly treated by CPAP which improves oxygenation to the inner ear leading to a reduction in hydropic distention of the endolymphatic system [7].

Besides MD, there have been some reports on other neurotological consequences of OSAS. Vestibular function had been demonstrated to be impaired in patients with OSAS [25, 26]. In 2010, Sowerby et al. first described a possible link between idiopathic dizziness, daytime somnolence, and sleep apnoea [27]. Kim et al. reiterated that sleep disturbance should be considered in patients with chronic subjective or nonspecific dizziness [28]. More recently, Foster and Machala reported that some OSA patients exhibited brief spells of nonpositional vertigo recurring throughout the day which responded to CPAP treatment [10]. OSAS has also been linked to sudden sensorineural hearing loss [29, 30]. However, in a large Korean study over 9 years, an association between OSAS and sudden sensorineural hearing loss could not be demonstrated [9].

## 4. Conclusion

The otologist/neurotologist must be mindful that some patients with MD may have concomitant OSA. Unless this comorbidity is treated, it may not be possible to control the symptoms of MD with standard medical therapies. Identification and successful treatment of OSA could potentially avoid the need for functionally destructive MD surgeries which are normally reserved for patients with uncontrolled disease. Symptoms suggestive of OSA must be proactively sought as these patients may not exhibit obvious phenotypic features of OSAS, especially in Asians.

## Figures and Tables

**Table 1:** AAO-HNS-amended (2015) diagnostic criteria for Meniere's disease.

*Definite*
(i) Two or more spontaneous episodes of vertigo, each lasting 20 min to 12 h
(ii) Audiometrically documented low- to midfrequency sensorineural hearing loss in 1 ear, defining the affected ear on at least 1 occasion before, during, or after 1 of the episodes of vertigo
(iii) Fluctuating aural symptoms (hearing, tinnitus, or fullness) in the affected ear
(iv) Not better accounted for by another vestibular diagnosis

*Probable*
(i) Two or more episodes of vertigo or dizziness, each lasting 20 min to 24 h
(ii) Fluctuating aural symptoms (hearing, tinnitus, or fullness) in the affected ear
(iii) Not better accounted for by another vestibular diagnosis

**Table 2:** Diagnostic criteria for vestibular migraine proposed by the Barany Society and the third International Classification of Headache Disorders (ICHD-3), 2012.

*Vestibular migraine*
(i) At least five episodes with vestibular symptoms of moderate^a^ or severe^b^ intensity, lasting 5 min to 72 h
(ii) Current or previous history of migraine with or without aura according to the International Classification of Headache Disorders (ICHD)
(iii) One or more migraine features with at least 50% of the vestibular episodes: (i) headache with at least two of the following characteristics: one-sided location, pulsating quality, moderate^a^ or severe^b^ pain intensity, and aggravation of routine physical activity; (ii) photophobia and phonophobia; (iii) visual aura
(iv) Not better accounted for by another vestibular or ICHD diagnosis

*Probable vestibular migraine*
(i) At least five episodes with vestibular symptoms of moderate or severe intensity, lasting 5 min to 72 h
(ii) Only one of criteria B and C for vestibular migraine is fulfilled (migraine history or migraine features during the episode)
(iii) Not better accounted for by another vestibular or ICHD diagnosis

^a^Usually interfere with daily activities. ^b^Usually prohibit daily activities.
